# Michelangelo Merisi da Caravaggio (1571–1610). Basket of Fruit (1596)

**DOI:** 10.3201/eid0912.AC0912

**Published:** 2003-12

**Authors:** Polyxeni Potter

**Affiliations:** * Centers for Disease Control and Prevention, Atlanta, Georgia, USA

**Figure:**
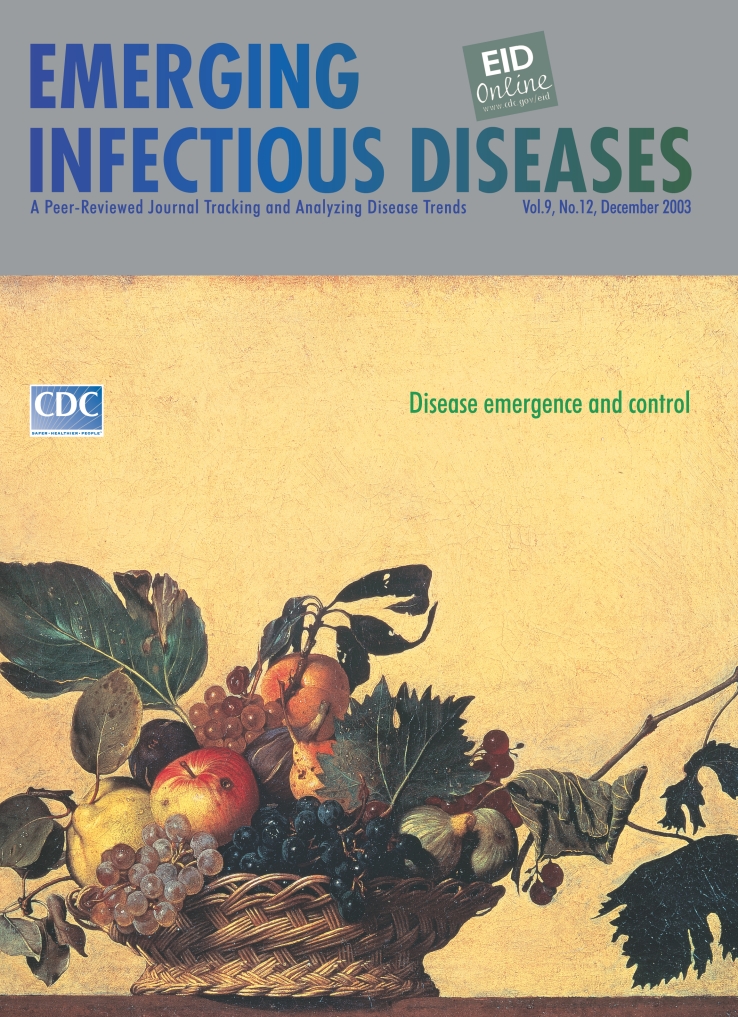
Michelangelo Merisi da Caravaggio (1571–1610). Basket of Fruit (1596) Oil on canvas, 45.92 cm x 64.46 cm Pinacoteca Ambrosiana, Milan, Italy

Born Michelangelo Merisi, Caravaggio was later renamed after his hometown in northern Italy, a practice not unusual in his day. His father, an architect and majordomo to the Marquis of Caravaggio, died of the plague when the artist was still young, leaving him under the protection of the art-loving marquis. Like many children of his day, he learned early how to grind pigments for painting, and soon he was apprenticed to a good studio in Milan. At 21, he moved to Rome, anxious, if not fully qualified, to compete in the capital’s bustling art world. This move to Rome began the tumultuous life journey of a man who changed the art of his day, had many followers (known as Caravaggists), and influenced future masters, from Rembrandt to Velázquez (*1*).

In Rome’s cosmopolitan art scene, the young Caravaggio found scant opportunity and slow recognition. Handicapped by his exuberance, fiery temper, and heightened artistic sensitivity, he was unable to cope with restrictions and authority. Brash, overbearing, and irascible, he became entangled in riotous brawls and walked the disorderly side of the capital. All the while, he painted mellow canvases overflowing with empathy, humanity, and compassion. Inventing a new, radical kind of realism, he populated his pictures with ordinary people, embracing their imperfections and weaknesses with a candor that many of his contemporaries mistook for vulgarity (*2*).

As if to decipher the contradictions and paradoxes of his own shadowy character, Caravaggio explored the interplay of light and dark, known in Italian as chiaroscuro. In an exaggerated theatrical style, he cast light selectively, adding drama to scenes, illuminating figures, and creating a poetic reality that was both earthy and mystical (*3*).

With uncanny immediacy, he painted potent images of beheadings and executions, perhaps anticipating the horror of his own punishment for unsavory behavior, not the least of which was killing his opponent during a tennis game. Arrested, imprisoned, pardoned, and constantly on the edge, Caravaggio continued to paint while living in exile for the last 4 years of his life. The disregard for limits that distinguished his work finally overcame his artistic promise. He died before age 40 of fever following one final, ironically mistaken, arrest.

During the early days of his tenure in Rome, unknown, unemployed, and unappreciated, Caravaggio painted religious images and baskets of fruit and flowers. Still-life painting, the domain of beginners since antiquity, ranked low on the hierarchical order of pictorial genres. Reduced to it by circumstance, Caravaggio elevated the genre to new heights, creating a European tradition that explored the “secret lives of objects” (*4*).

“I put as much effort in painting a basket of flowers as I do in painting human figures,” Caravaggio told an early patron (*3*). Taking the first step toward abstract art, he allowed space to be defined by objects—their form, angle, solidity, and composition. Instead of idealizing nature, as the classicists advocated, he painted its imperfections, investing objects with uniqueness and personality. Instead of centering them on the canvas, he thrust them provocatively in the viewer’s face, demanding attention and participation.

“I would have…hung a similar basket next to it but as no one was able to attain its incomparable beauty and excellence, it remained alone,” 17th-century cardinal Federico Borromeo said of Caravaggio’s Basket of Fruit (*5*), on this month’s cover of Emerging Infectious Diseases.

This “incomparable” basket, probably painted over a number of days, has a weathered familiarity, its ripened contents settled, its branches jutting stiffly out the edge. Though representing tradition and plentitude, the fruit is past its prime. Only the tart quince seems to be holding firm. Soft and lusterless, the apple is pockmarked and flawed. The grapes hang heavy, their translucent skin spotted and brown against the plump figs. The leaves, colors fading, edges curling and snarled, are brittle and crinkly. Yet, against an abstract backdrop of brilliant gold leaf, this laden basket exudes comfortable elegance, tangible beauty, graceful maturity.

Caravaggio’s painting is not just a lyrical composition of forms. Engaging the senses in virtual abundance, which like life itself is all too ephemeral, the basket comments on the complexity and vanity of nature. Defying the moment of creation, the diverse image spans instead the life of the fruit, commenting on its inevitable decay. The blemishes, intentional and central to the theme, are not brought on by precipitous mishap but by nature. Uncontrolled environment (temperature, moisture, microorganisms) has disrupted the fruit’s normal physiology, devitalizing the skin, allowing invasion of pathogens, and promoting decomposition.

In our world, as in Caravaggio’s, where light and darkness, beauty and horror, pleasure and danger are constantly at play, survival depends on keeping the elements of nature in balance, constantly tracking their course, monitoring their moves, and checking their excesses. Left untended and uncontrolled, nature’s elements will thrive to unfair advantage, mutate to our detriment, and travel to our doorstep. In formerly out-of-the-way places like Mongolia, where control efforts have not always kept pace, old scourges (tuberculosis, brucellosis, plague, tularemia) maintain their insidious hold, a blemish on world health and a threat to balance and control.
